# A multiscale approach to detect selection in nonmodel tree species: Widespread adaptation despite population decline in *Taxus baccata* L

**DOI:** 10.1111/eva.12838

**Published:** 2019-07-19

**Authors:** Maria Mayol, Miquel Riba, Stephen Cavers, Delphine Grivet, Lucie Vincenot, Federica Cattonaro, Giovanni G. Vendramin, Santiago C. González‐Martínez

**Affiliations:** ^1^ CREAF Cerdanyola del Vallès Spain; ^2^ Univ. Autònoma Barcelona Cerdanyola del Vallès Spain; ^3^ Centre for Ecology and Hydrology Edinburgh Penicuik UK; ^4^ Department of Forest Ecology and Genetics, Forest Research Centre INIA‐CIFOR Madrid Spain; ^5^ Sustainable Forest Management Research Institute, INIA‐University of Valladolid Madrid Spain; ^6^ UNIROUEN, IRSTEA, ECODIV Normandie Univ. Rouen France; ^7^ Instituto di Genomica Applicata (IGA) Udine Italy; ^8^ Institute of Biosciences and Bioresources, Division of Florence National Research Council Sesto Fiorentino Italy; ^9^ UMR BIOGECO, INRA Univ. of Bordeaux Cestas France

**Keywords:** adaptation, demographic decline, English yew (*Taxus baccata*), environmental association, polygenic adaptation, single nucleotide polymorphism

## Abstract

Detecting the molecular basis of local adaptation and identifying selective drivers is still challenging in nonmodel species. The use of purely population genetic approaches is limited by some characteristics of genetic systems, such as pleiotropy and polygenic control, and parallel evidence from phenotypic‐based experimental comparisons is required. In long‐lived organisms, the detection of selective pressures might also be precluded by evolutionary lag times in response to the environment. Here, we used the English yew to showcase an example of a multiscale integrative approach in a nonmodel species with limited plant and genomic resources. We combined information from two independent sources, phenotypes in a common environment and genomic data in natural populations, to investigate the signature of selection. Growth differences among populations in a common environment, and phenological patterns of both shoot elongation and male strobili maturation, were associated with climate clines, providing evidence for local adaptation and guiding us in the selection of populations for genomic analyses. We used information on over 25,000 SNPs from c. 1,200 genes to infer the demographic history and to test for molecular signatures of selection at different levels: SNP, gene, and biological pathway. Our results confirmed an overall demographic history of population decline, but we also found evidence for putative local adaptation at the molecular level. We identified or confirmed several candidate genes for positive and negative selection in forest trees, including the *pseudo‐response regulator 7* (PRR7), an essential component of the circadian clock in plants. In addition, we successfully tested an approach to detect polygenic adaptation in biological pathways, allowing us to identify the *flavonoid biosynthesis* pathway as a candidate stress‐response pathway that deserves further attention in other plants. Finally, our study contributes to the emerging view that explaining contemporary standing genetic variation requires considering adaptation to past climates, especially for long‐lived trees.

## 1 INTRODUCTION

Understanding adaptive genetic responses to climate is crucial for preserving biological diversity and predicting future evolutionary responses, particularly for many forest trees that are keystone species within the ecosystem. In the past few years, the increase in forest tree genomic resources, coupled with the development of novel analytical approaches, has contributed to understand how adaptive and neutral processes have shaped genetic variation of tree species (Holliday et al., 2017). Nevertheless, there are some major challenges ahead. On the one hand, from a methodological point of view, the power to detect loci under selection with commonly used approaches (e.g., *F*_ST_‐outlier detection or environmental association) is variable depending on the sampling effort and the number of environmental variables included (Ahrens et al., 2018). On the other hand, and despite the considerable effort made to discern between selective and purely demographic processes, these methods are still prone to high false‐positive rates (Lotterhos & Whitlock, 2014; Villemereuil, Frichot, Bazin, Francois, & Gaggiotti, 2014). Moreover, accounting for the underlying population structure to reduce false positives may end up removing true adaptive variants when population structure and environmental drivers of adaptation are aligned, particularly when the trait is polygenic and/or the selection signature is weak (Ahrens et al., 2018). Some alternative strategies have been applied to circumvent these problems. For example, in a study investigating convergent adaptation to climate in distantly related conifers (*Pinus contorta* and *Picea glauca* complex), the authors did not correct by population structure but assumed that significant environmental associations shared across species could not have arisen from purely neutral processes (Yeaman et al., 2016). Similar approaches can then be applied to complement conventional methods of studying adaptation with genomic data.

From a biological point of view, an additional and particularly important limitation is that most methods are designed to detect the signature of selection at individual loci. However, many adaptive traits are probably governed by multiple loci, each contributing only modestly to the phenotype, and adaptation is expected to take place by selection on standing variation at many loci simultaneously (Pritchard, Pickrell, & Coop, 2010). Consequently, in model species like humans, an increasing number of studies have shifted from focusing on single genes to polygenic approaches based on biologically meaningful groups of genes that underpin processes such as metabolic pathways (e.g., Daub et al., 2013; Daub, Moretti, Davydov, Excoffier, & Robinson‐Rechavi, 2017; Berg & Coop, 2014; Foll, Gaggiotti, Daub, Vatsiou, & Excoffier, 2014). To date, however, few studies have applied multilocus approaches to investigate the mechanisms underlying local adaptation in forest trees (but see Csilléry et al., 2014), and even fewer in the case of nonmodel tree species with limited available genomic resources.

The characteristics of genetic systems, such as pleiotropy and polygenic control, place limits on the use of purely population genetic approaches to infer local adaptation, known as “reverse ecology” (Li, Costello, Holloway, & Hahn, 2008). To successfully use genomic analyses to detect the molecular basis of local adaptation and identify selective drivers require support from organism‐based approaches (Tiffin & Ross‐Ibarra, 2014). Unfortunately, in many cases, genomic analyses targeting adaptation often do not consider relevant additional information on phenotypic traits (Barrett & Hoekstra, 2011; Villemereuil, Gaggiotti, Mouterde, & Till‐Bottraud, 2016). In forest trees, common garden approaches have been widely used to study adaptive evolution in traits such as growth, wood properties, and cold‐hardiness, but most experiments have been restricted to species and traits of economic value. Extending the use of experiments to species of noneconomic interest, especially those with contrasting life‐history traits, would improve our understanding of the multiple factors that influence adaptation in forest trees. However, performing well‐designed, replicated common garden experiments on nonmodel species, or those not considered economically important, is certainly a challenge in view of the general lack of funding. This is even more problematic when, due to the species’ distribution and population characteristics, access to plant material is limited.

Finally, association of climatic variables with potentially adaptive molecular variation has usually been restricted to using contemporary environmental data. However, in tree populations, time lags may develop in genotype–environment associations and persist for many generations as a consequence of long generation times and late reproductive age. Therefore, genetic variation in long‐lived species might not be in equilibrium with current environments but rather be associated with past ones (e.g., Gugger, Ikegami, & Sork, 2013; Epps & Keyghobadi, 2015; Mayol et al., 2015). Exploring the association of genetic variability with environment through time is essential to dissect the relative contribution of past and present climates to the patterns observed. This can be addressed by applying a temporal dimension to population genomic approaches: Loci conferring adaptation to past environments that are selectively neutral in modern‐day conditions would be expected to show significant associations with paleoenvironments, while the inverse would be expected for loci conferring adaptation to current climate (Lafontaine, Napier, Petit, & Hu, 2018).

In this study, we used the English yew (*Taxus baccata* L.) to showcase an example of an integrative approach in a nonmodel species with limited plant and genomic resources, taking into consideration the issues raised above concerning the study of local adaptation. We first addressed the phenotypic variability and potential adaptive responses of trees from a reduced but climatically heterogeneous part of the species range (Iberian Peninsula) growing in a common environment. From the results obtained, we designed an independent geographic sampling to enable a rangewide genomic analysis. Taking advantage of the recently available *T. baccata* transcriptome (Olsson et al., 2018), we designed a gene capture approach for ~1,200 genes, including known candidate genes for climate adaptation in forest trees. Then, we inferred the demographic history of populations and applied several methods to investigate the signature of selection at single‐locus (SNP) and multilocus (gene, biological pathway) levels. Since neutral genetic diversity at the whole distribution level bears the signature of potential adaptation to present and past environments (Mayol et al., 2015), we used climatic information from the present, the last glacial maximum and the last interglacial periods to evaluate the relative importance of current and past climatic conditions on patterns of genetic variation.

English yew is a slow‐growing and long‐lived dioecious gymnosperm presently found in a variety of habitats throughout most of the European continent. Despite its wide distribution, it often forms small and isolated populations, mainly in the Mediterranean area (Thomas & Polwart, 2003). Most natural or seminatural populations are not easily accessible, and obtaining large amounts of seed per population and female tree is a limiting issue for adequate replication in provenance trials. Furthermore, conditions for seed germination and storage are highly restrictive, and dormancy is highly variable (Thomas & Polwart, 2003). In contrast to other gymnosperms, gene flow among English yew populations is limited, and genetic diversity is highly structured both at the local (Dubreuil et al., 2010) and the regional scale (Chybicki, Oleksa, & Kowalkowska, 2012; González‐Martínez et al., 2010). Its demographic history seems to be mostly characterized by a strong and continuous reduction of the effective population size since the last interglacial, as suggested by palaeoecological records (e.g., Turner, 2000; de Beaulieu et al., 2001; Koutsodendris et al., 2010) and molecular data, at least for populations from the Iberian Peninsula (Burgarella et al., 2012).

The small populations typical of English yew are predicted to have restricted capacity to adapt to environmental change because enhanced genetic drift opposes the effect of natural selection (Wright, 1931) and increases genetic load (i.e., fixed deleterious mutations; Whitlock, 2000). However, some empirical studies have suggested that divergent selection on phenotypic traits might also be enhanced in small, fragmented populations (Willi, Van Buskirk, Schmid, & Fischer, 2007). Consistent with these results, the signature of ongoing or recently completed selection was found in some of the genes involved in taxol biosynthesis in Iberian yew populations, despite a clear signature of recent (2,000–3,000 generations) bottlenecks (Burgarella et al., 2012). Our goals were thus (a) to investigate whether other potentially adaptive traits, such as those related to growth and phenology, show evidence of local adaptation in Iberian populations of the species, (b) to assess the role of local adaptation at the rangewide scale in a species characterized by demographic bottlenecks, and (c) to determine the contribution of present and past environments to the observed patterns of variation and the most likely environmental drivers underlying them.

## 2 MATERIALS AND METHODS

### 2.1 Phenotypic variability in a common garden

**Figure 1:**
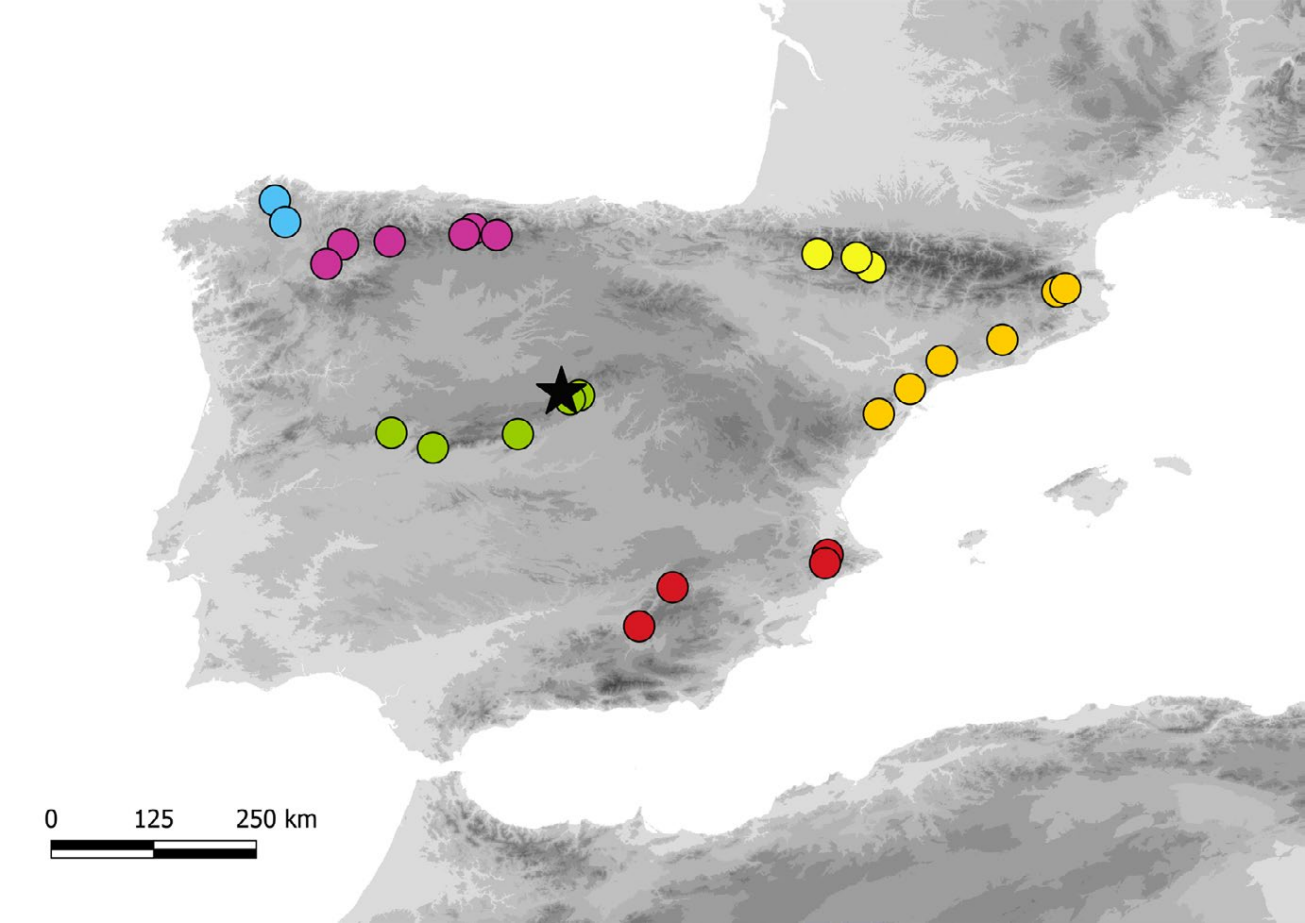
Geographic distribution of the 26 natural populations of *Taxus baccata* planted in the Valsaín clonal bank (see details in Table S1). Colors for populations indicate different bio‐geographic regions: Atlantic Region (blue dots), Cantabrian Mountains (dark pink dots), Catalan Coastal Range (orange dots), Central System (green dots), Pyrenees (yellow dots), and Sub‐Baetic System (red dots). The location of Valsaín experimental site is indicated by a black star

The analysis of phenotypic variation was carried out in the Valsaín clonal bank, located in central Spain (Segovia, 40º54′38″N, 04º00′45″W, 1,136 m a.s.l; Figure 1). This unique clonal *T. baccata* collection has been progressively built since 1992 for ex situ conservation of genetic resources in the Iberian Peninsula. Plant material (genets) was collected in 26 native populations from one‐year‐old shoots located on terminal branches. Due to the patchy distribution of the species, populations are easily defined as a group of reproducing individuals growing along ravines or hillsides and readily identified in the landscape. Clones were propagated through stem cuttings in growth chambers and later transferred outdoors. Plants were planted 2–3 m apart in a row and column layout without following any particular randomized design, replaced when dead, and watered if needed during summer. After accounting for ramets failing to establish, the available plant material included a total of 254 uneven aged (10–15 year old) clones (individual plants) belonging to 108 genets (1–9 genets per population) distributed across six contrasted geographic regions in the Iberian Peninsula (Figure 1, Table S1).

First, we measured shoot growth, which is often considered as a fitness proxy in forest trees (e.g., Alía, Chambel, Notivol, Climent, & González‐Martínez, 2014). Since young yew trees tend to grow as a shrub, individual growth was estimated as the volume (diameter^2^ * length, in mm^3^) of the leading current‐year shoot located in the two dominant (longest) stems in four consecutive years (2009–2012). We used the average value of both leading shoots as the response variable. According to the number of replicates available, the variability in growth was assessed using a Gaussian mixed model with Population, Sex, Year, and Population by Year interaction as categorical fixed factors. We used stem length at the beginning of our follow‐up study (2009) as a covariate. Genet and individual within genet were treated as random factors. Stem length and shoot volume were log‐transformed after residual analyses.

Second, we investigated the phenology of shoot elongation and male strobili maturation from 2010 to 2012. Growth phenology was assessed by comparing the ratio of shoot length produced before the onset of summer drought (May‐June) to the total shoot length attained at the end of the growing season (October‐November). Measures of length were taken on the leading current‐year shoot of two dominant stems per individual and averaged before analyses. Male strobili maturation was assessed in two consecutive samplings (early and mid/late March) on tagged apical shoots and was estimated as the proportion of strobili shedding pollen using pooled data from both samplings. In both cases, we used mixed models to determine the effects of Population, Sex and Year, using genet and individual within genet as random effects. In the analysis of shoot elongation, we also tested for Sex by Year and Population by Year interactions. Models were conducted on the original scale assuming a Gaussian distribution of residuals. Strobili maturation was assessed in a subsample of 21 populations. Since several individuals and genets failed to reproduce every year, we performed 2 analyses. In the first, we used 14 populations allowing us to test for the effect of Population, Year, and their interaction. In the second, we tested only for Population and Year main effects using all populations. Models of the proportion of mature strobili were run assuming binomial random errors.

All mixed models were performed in R v. 3.4.4 (R Core Team, 2018) using the *lme4* package (Bates, Maechler, Bolker, & Walker, 2015). For models with Gaussian errors, we report *F* tests based on Kenward–Roger's approximation of the degrees of freedom. Reported *P*‐values for mixed models with binomial type errors are based on likelihood ratio tests. Comparisons of mean values across groups were performed using the *emmeans* package (Length, 2018). Estimated marginal means for each Population were used to determine associations with climate variables. Monthly records of temperature (mean, maximum and minimum) and precipitation were obtained from the Digital Climatic Atlas of the Iberian Peninsula (://opengis.uab.es/wms/iberia/en_index.htm; Ninyerola, Pons, & Roure, 2005). From these data, we calculated monthly temperature range and averaged seasonal values for climatic variables: winter (December‐February), spring (March‐May), summer (June‐August), and fall (September‐November). For the same variables and periods, we also included estimates of calibrated climatic distance (Gower's distance) between the location of each source population and the average values at the common garden site recorded during the study period (see Rutter & Fenster, 2007).

### 2.2 Sampling for molecular analyses and DNA extraction

**Figure 2:**
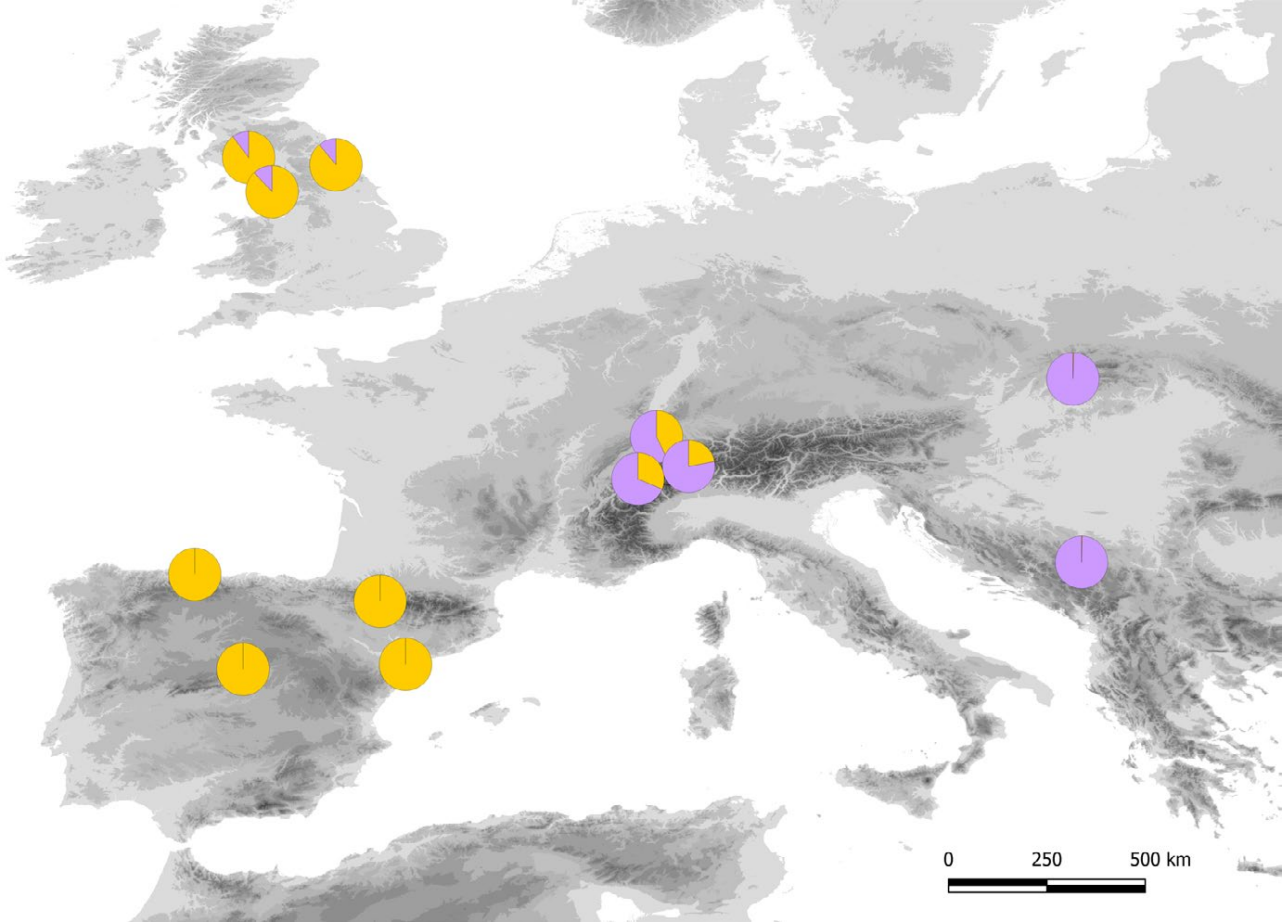
Location of the European populations of *Taxus baccata* sampled for the gene capture experiment. Pie charts show the proportion of gene pool membership (*K* = 2) by population inferred from 25,726 SNPs with faststructure. See Table S2 for details on populations

Phenotypic variability in the common garden suggested a potential cline of adaptation from cold, inland, continental climates to milder, temperate, and coastal ones (see Results). Based on these results, we sampled 12 populations throughout the European distribution of English yew spanning a range of variation from cold continental to milder temperate conditions (Figure 2, Table S2). Since we aimed to test for the effects of both present and past climatic conditions, populations were sampled from locations where suitable environments for yew persistence were present during the last glacial maximum (LGM, *c.* 21,000 year BP) and the last interglacial (LIG, *c.* 120,000–140,000 year BP), in agreement with Mayol et al. (2015). Ten individuals were sampled in each population, resulting in a total sample size of 120 individuals. Total DNA was isolated from 50 to 100 mg of dry leaf material using the Invisorb^®^ DNA Plant HTS 96 Kit (STRATEC Molecular GmbH, Berlin, Germany).

### 2.3 Selection of genes for sequencing

We used two main strategies to select candidate genes. First, we reviewed the literature (up to 2012) focused on the characterization of loci putatively involved in the adaptation of tree species to environment (e.g., cold, drought, pathogens, phenology) and obtained a list of 344 genes of interest (Table S3). Then, we retrieved the reference sequences from these publications and aligned them to the *T. baccata* transcriptome (Olsson et al., 2018) using BLAST (Altschul, Gish, Miller, Myers, & Lipman, 1990). Positive results were obtained for 273 genes > 450 bp, which were selected for subsequent analyses.

Second, the transcriptomes of *T. baccata* (Olsson et al., 2018), *T. cuspidata* (Lee et al., 2010), *T. wallichiana* (Hao, Ge, Xiao, Zhang, & Yang, 2011), and *Picea sitchensis* (European Bioinformatics Institute, TSA project GACG01000000 data at http://www.ebi.ac.uk/) were used to compute *K*_a_/*K*_s_ and detect additional potential candidates under moderate/high positive evolution (*K*_a_/*K*_s_ > 0.5 for comparisons with *T. cuspidata* and *T. wallichiana*; and *K*_a_/*K*_s_ > 0.3 for those with *P. sitchensis*). From this approach, we obtained 345 candidate genes with > 450 bp length and functional annotation available, so the final number of candidate genes obtained from literature and sequence divergence ratios was 618 (797,452 bp).

Finally, we completed our design including 516 genes with > 450 bp length for which functional annotation was available (753,183 bp), despite not being selected as candidates initially by any of the two approaches. Thus, the final number of genes included in the study was 1,134 (1,550,635 bp).

### 2.4 Probe design, library construction, and targeted sequencing

Selected genes were sequenced in the 120 yew trees by a sequence capture approach using a custom SeqCap EZ design (Roche NimbleGen Inc, Basel, Switzerland) followed by next‐generation sequencing of captured regions on an Illumina platform. Probes were designed by Roche Tech‐Support in Madison (WI, USA) starting from the selected 1,134 gene sequences (about 1.6 Mbp of target sequence in total). Library preparation and targeted sequencing were outsourced to IGA Technology Services (Udine, Italy). Libraries were constructed by using KAPA DNA Library Preparation Kit (Roche, NimbleGen Inc.) following the manufacturer's protocol and enrichment performed using NimbleGen solution‐based SeqCap EZ probe libraries kit. Cluster generation, template hybridization, isothermal amplification, linearization, blocking and denaturization, and hybridization of the sequencing primers were then performed on Illumina cBot and flow cell HiSeq SBS V4 50 cycle kit, loaded on HiSeq 2500 Illumina sequencer producing 50 bp single reads. The CASAVA version of the Illumina Pipeline 1.8.2 was used for base calling and de‐multiplexing. Adapters were masked using *cutadapt* (Martin, 2011). Masked and low‐quality bases were filtered using *erne‐filter* (Del Fabbro, Scalabrin, Morgante, & Giorgi, 2013).

The GATK pipeline was used for SNP calling, following GATK best practices for SNP filtering (–filterExpression “MQ0> = 4 && ((MQ0/(1.0 * DP)) > 0.1) || DP < 10.0 || Q < 50.0 || QD < 1.5 || FS > 60.0”), which allowed the initial identification of 52,904 SNPs. SNP data were further filtered using VCFtools v0.1.14 (Danecek et al., 2011) and vcffilter (part of vcflib C++ library) for average coverage across all samples (between 20 and 250) and allele balance (between 0.3 and 0.7 for the heterozygous calls). Only polymorphic biallelic SNPs with fewer than 15% missing data were kept. This stringent filtering reduced the SNP dataset to 36,028 SNPs. This dataset was additionally filtered by removing SNPs that showed a significant heterozygote excess in ≥4 populations to minimize the impact of putative paralogous loci on data analysis, leaving a final dataset of 25,726 SNPs spanning 1,128 genes (six out of the initial 1,134 genes were discarded because of low sequence quality).

### 2.5 Genetic diversity, population structure, and demography

Overall nucleotide diversity (*π*), the efficacy of selection evaluated as the ratio of nonsynonymous to synonymous nucleotide diversity (*π*_a_/*π*_s_), and neutrality tests (Tajima's *D,* and Fu & Li's D* and F*) were computed using mstatspop (https://bioinformatics.cragenomica.es/numgenomics/people/sebas/software/software.html) on concatenated sequence files, both considering all populations together and separately for distinct geographic regions (Bosnia‐Herzegovina, Iberian Peninsula, Slovakia, Switzerland and United Kingdom, Table S2). Average inbreeding coefficients (*F*) and pairwise relatedness (unadjusted *A_jk_* statistic, Yang et al., 2010) were computed using VCFtools v0.1.14 (Danecek et al., 2011). Additionally, relatedness between pairs of individuals within each population was computed to discard the potential inclusion of replicated individuals (clones) in the samples.

Population genetic structure was analyzed using the program faststructure (Raj, Stephens, & Pritchard, 2014), from *K* = 1 (i.e., no structure) to *K* = 12 (the number of localities in our sample). Runs were repeated three times, and averaged *Q*‐values (i.e., the individual assignment probability to each of the *K* groups) were used to draw pie charts in Figure 2. In a previous study, Burgarella et al. (2012) showed strong demographic decline (based on nuSSRs) in yew populations from the Iberian Peninsula. To confirm or refute these results at a wider geographic range, we assessed effective population size over time using the stairway plot approach on unphased SNP data (version 2 beta; Liu & Fu, 2015). We assumed a per‐generation mutation rate of 1.4 × 10^–8^ considering per‐year mutation rates for conifers in Chen, Uebbing, et al. (2012) and a generation time for English yew of 50 years (Thomas & Polwart, 2003). Median estimates of the population size and confidence intervals were estimated with the built‐in bootstrap function using 200 replicates of the input file.

### 2.6 Detecting signatures of selection at SNP, gene, and pathway level

We combined several approaches to investigate the contribution of natural selection to genetic adaptation in English yew. First, we searched for signals of selection at the single‐locus (SNP) level using two environmental association approaches that take into account population genetic structure to avoid false positives: bayescenv (Villemereuil & Gaggiotti, 2015) and baypass (Gautier, 2015). For each population, we downloaded the climatic information available at the WorldClim database (Hijmans, Cameron, Parra, Jones, & Jarvis, 2005) for the present (PRE, *c*. 1950–2000), the last glacial maximum (LGM, *c*. 21,000 year BP) and the last interglacial (LIG, *c*. 120,000–140,000 year BP) periods. We obtained data for eight climatic variables for each period: annual mean temperature, temperature seasonality (*standard deviation*), maximum temperature of the warmest month, minimum temperature of the coldest month, annual precipitation, precipitation of the wettest month, precipitation of the driest month, and precipitation seasonality (coefficient of variation). bayescenv was run for each of the standardized climatic variables with general default parameters. After 20 pilot runs of 2,000 iterations and a burn‐in of 50,000 iterations, 5,000 MCMC samples were taken with 10 steps between each sample. For baypass runs, standardized variables were run using default parameters under the standard model (STD in Gautier, 2015). We retained all significant SNP–climate associations with *Q*‐value < 0.05 (bayescenv)  and/or Bayes Factor > 30 (baypass).

Second, we searched for genes with a high proportion of SNPs being correlated to climatic variables using a similar approach to the method proposed by Yeaman et al. (2016). For each SNP, we calculated Spearman's rank correlations between allele frequency and the 24 variables (8 climatic variables × 3 periods), and retained all SNPs with significant correlations at *p* < 0.01. For each gene, we then counted the total number of SNPs (*n*) and the number of SNPs with significant climate correlations (*a*). Then, to identify candidate genes potentially under selection, we compared the number of SNPs related to climate in each gene to the 0.9999 quantile of its binomial expectation, considering the expected frequency of SNPs related to climate per gene to be *P* = ∑*_i_a_i_*/*n_i_* (summation over *i* genes), and calculating *P* separately for each climatic variable. Following Yeaman et al. (2016), genes with zero outliers were excluded from the calculation of *P*. Finally, to control for false positives and produce a more robust test, we combined the results of this approach with those obtained with the SNP analyses and retained only those genes that also had at least two different SNPs that were significantly associated with the same climatic variable in bayescenv and/or baypass. Candidate genes were annotated using BLASTX searches against the *Picea abies* genome at ConGenIE public database (://www.congenie.org; Nystedt et al., 2013), using a minimum threshold of 50% of sequence identity and E‐value < 1E‐50. The functional annotation of candidate genes was then based on the associated *A. thaliana* description for each of the best hits provided with the Norway spruce reference genome sequence.

Third, we used a complementary, independent, procedure to investigate the potential joint effect of selection acting on multiple loci (polygenic adaptation) instead of on single genes. This procedure was, in turn, independent of the environmental variables chosen, allowing the detection of potential signatures of selection in response to other drivers apart from climate. For this purpose, we used KEGG Automatic Annotation Server to map our 1,128 genes and retained 75 pathways that included at least five genes. We refined this pathway list by retaining only known pathways in plants, most of them having an *Arabidopsis thaliana* reference map. The final dataset thus consisted of 390 distinct genes that were mapped on 60 pathways, of which eight were plant‐specific. Then, for each sequence‐based statistic obtained with mstatspop  (*π*, *π*_a_/*π*_s_, Tajima's *D*, and Fu & Li's D* and F*), we performed a gene set enrichment approach as in Daub et al. (2013), using polysel gene set enrichment pipeline freely available from ://github.com/CMPG/polysel. Briefly, for each statistic, we calculated the SUMSTAT score (Tintle, Borchers, Brown, & Bekmetjev, 2009), which is simply the sum of the values for that statistic of all genes in a given pathway, and compared it to a null normal distribution of 100,000 random sets with the same size as the original pathway. The null distribution was created with a sequential random sampling method (Ahrens & Dieter, 1985). Following Daub et al. (2013), a “pruning” approach was applied to avoid any redundancy between gene sets (see also polysel manual). Briefly, the genes belonging to the highest scoring gene set were removed, iteratively, from the remaining gene sets and the testing procedure was re‐run until no gene sets large enough to be tested were left. When following this procedure, tests are not independent and biased toward low *P*‐values (i.e., only the higher scoring gene sets remain after pruning), and thus, we estimated the false discovery rate (FDR) empirically using 300 iterations.

## 3 RESULTS

### 3.1 Adaptive variation at the phenotypic level

**Figure 3:**
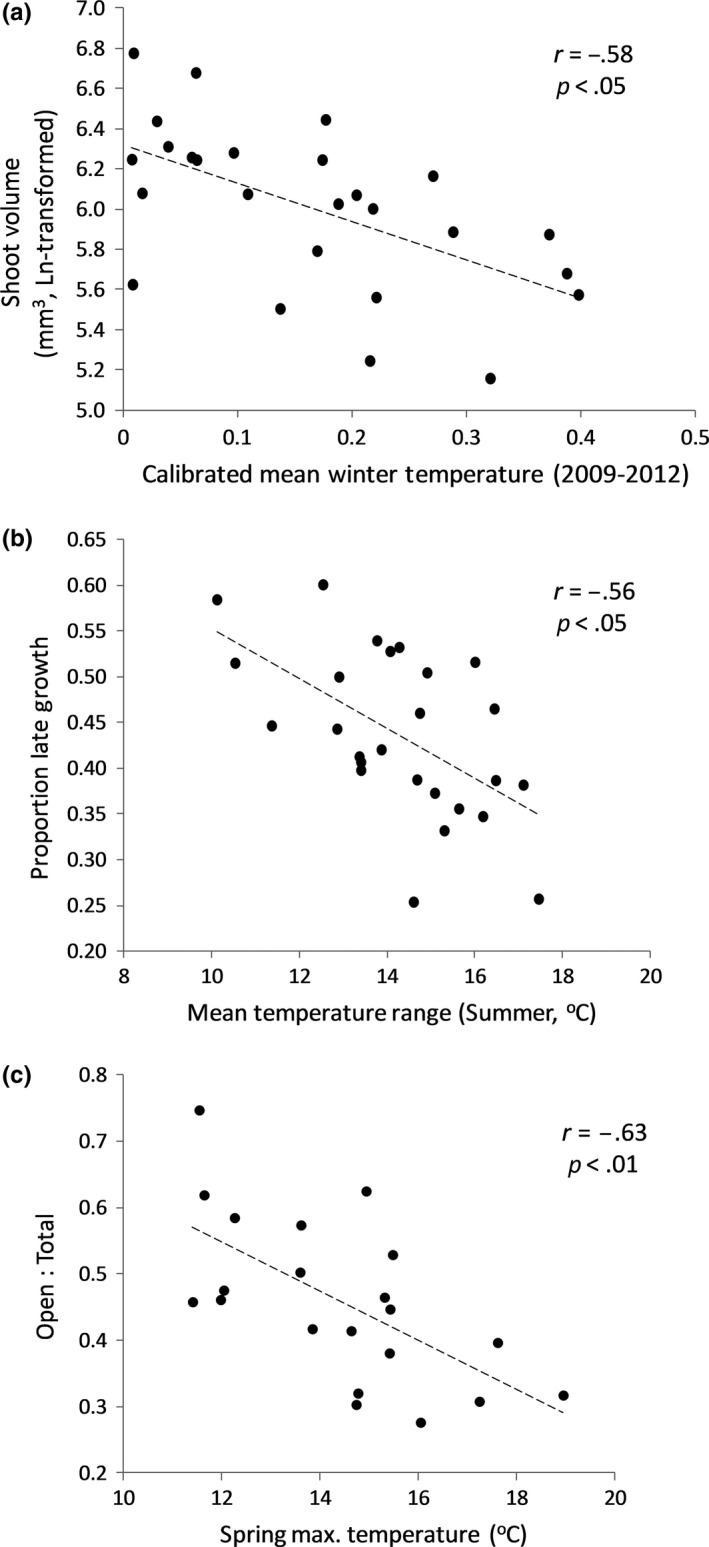
Phenotypic variation measured in the common garden (Valsaín clonal bank) and its correlation with climate. (a) Correlation between mean population shoot growth (volume, mm^3^) and calibrated winter temperature (Gower's distance); (b) correlation between the mean proportion of late (summer‐fall) shoot growth (in length) and mean temperature range in summer; (c) correlation between male strobili maturation (estimated as the proportion of open strobili shedding pollen relative to the total number of strobili) during the reproduction peak and spring maximum temperature. Models are based on estimated marginal means, see Materials and Methods for details

The analysis of growth showed significant differences between sexes (*F*_1, 66.03_ = 4.65; *p* < 0.05). Males showed higher annual growth than females (Figure S1). We also found differences among populations across years (Population by Year interaction: *F*_75, 650.02_ = 1.85; *p* < 0.001). However, relative differences among populations were quite consistent across years (Figure S2). We also found significant negative correlations between mean population growth and calibrated mean and maximum winter temperatures (Pearson's *r* = −0.58 and −0.49, respectively; *p* < 0.05; Figure 3a), suggesting local adaptation for growth to temperatures experienced during the colder seasons. Removing the populations with the lowest level of replication (those with less than three genets) produced the same results and improved the relationship between growth and calibrated environmental variables (e.g., *r* = −0.74 and *p* < 0.001 for mean winter temperature).

Significant differences among populations were also found in phenological patterns of shoot elongation and maturation of male strobili. The proportion of late shoot growth (from early summer to late fall) varied among populations and between sexes, but such differences were also dependent on the year considered (Population by Year interaction: *F*_50, 389.97_ = 1.89; *p* < 0.001; Sex by Year interaction: *F*_2, 391.32_ = 4.49; *p* < 0.05). However, differences among populations and sexes were highly consistent across years (Figures S3 and S4, respectively). Compared to males, higher values of late shoot growth in females suggest more protracted and slower growth in females. Patterns of variation in shoot growth phenology were significantly associated with temperature range during summer and fall (Pearson's *r* = −0.56 and −0.51, respectively; *p* < 0.05; Figure 3b). This relationship suggests that yew trees from more continental sites or located at higher elevations, with largest diurnal temperature range, started growth earlier and/or grew faster. The analysis of the variability in male strobili maturation (using a subset of populations, see Materials and Methods) showed a significant interaction between population and year (*χ* ^2^ = 476.64, *df* = 26, *p* < 0.001), although the overall pattern of differentiation among populations was also similar across years (Figure S5). Differences among years and populations in strobili phenology were also significant when considering all available populations (Year: χ^2^ = 1,770.5, *df* = 2, *p* < 0.001; Population: χ^2^ = 44.64, *df* = 20, *p < *0.01). We also found a significant negative correlation between strobili maturation and temperature, particularly mean and maximum temperature during winter and spring (Pearson's *r*= −0.64 to −0.61, *p* < 0.01 in all cases; Figure 3c), with male strobili maturing earlier in those genets from colder populations.

### 3.2 Genetic diversity, population structure, and demography

Overall synonymous nucleotide diversity (*π*_s_ = 0.00897) and efficacy of selection (*π*_a_/*π*_s_ = 0.308; Table 1) were similar to those found in other outcrossing long‐term perennial plants (reviewed in Chen, Glemin, & Lascoux, 2017). At the regional level, Slovakia and Bosnia‐Herzegovina (Eastern gene pool, see below and Mayol et al., 2015) showed a lower proportion of polymorphic sites, probably because the smaller number of individuals sampled in these regions (Table 1). Nucleotide diversity was consistently lower at southern latitudes, that is, Bosnia‐Herzegovina and the Iberian Peninsula. None of the individuals sampled was suspicious to be replicated due to clonality, since high values of pairwise relatedness (>0.6) within each population were restricted to the comparisons of an individual with itself (Table S4).

**Table 1:** Genetic diversity, neutrality test, and inbreeding statistics. Main statistics are given at the species level and separately for the different *Taxus baccata* geographic regions included in this study. *π*_a_/*π*_s_: efficacy of selection as evaluated by the ratio of nonsynonymous to synonymous *π* (Tajima, 1989); *TajD*: Tajima's *D* (Tajima, 1989); FuD*: Fu & Li's D* (Fu & Li, 1993); FuF*: Fu & Li's F* (Fu & Li, 1993); *F*: average inbreeding coefficient; *Related*: average pairwise relatedness statistic (unadjusted *A_jk_*) based on the method of Yang et al. (2010); values around zero indicate unrelated individuals

Geographic region	*N*	L (bp)	Polymorphic sites		Nucleotide diversity^a^ (*π*)				Neutrality test stats			Inbreeding	
			Number	%	All	Syn	Non‐syn	*π*_a_/*π*_s_	*TajD*	FuD*	FuF*	*F*	*Related*
Iberian Peninsula	40	1,472,324	20,680	80.38	3.97	8.00	2.53	0.316	1.09	0.90	1.10	−0.033	−0.023
United Kingdom	30	1,472,125	21,302	82.80	4.16	8.49	2.63	0.310	0.99	0.99	1.11	−0.107	−0.030
Switzerland	30	1,472,055	20,233	78.65	4.14	8.40	2.63	0.313	1.19	1.00	1.19	−0.101	−0.030
Slovakia	10	1,471,260	19,363	75.27	4.30	8.68	2.71	0.312	0.53	0.54	0.57	−0.178	−0.088
Bosnia‐Herzegovina	10	1,470,875	16,879	65.61	3.95	7.99	2.54	0.318	0.76	0.68	0.74	−0.091	−0.087
All	120	1,473,550	25,726	100	4.37	8.97	2.76	0.308	1.27	1.69	1.41	−0.086	−0.008

L, length of sequences; N, sample size.

Nucleotide diversity per site × 10^−3^; All: all sites, including coding regions (CDS), introns, and intergenic sequences; Syn: synonymous sites; Non‐syn: nonsynonymous sites.

Population genetic structure showed an optimal partition into two genetic pools located at the eastern (Bosnia‐Herzegovina, Slovakia) and western (Iberian Peninsula, United Kingdom) parts of the distribution (Figure 2), with a contact admixture zone in central Europe (Switzerland). All the additional partitions (*K* = 3–12) produced the same results, that is, the separation of Swiss populations as a third independent pool and, in some instances, Bosnia‐Herzegovina and Slovakia were split into two different genetic pools (data not shown).

**Figure 4:**
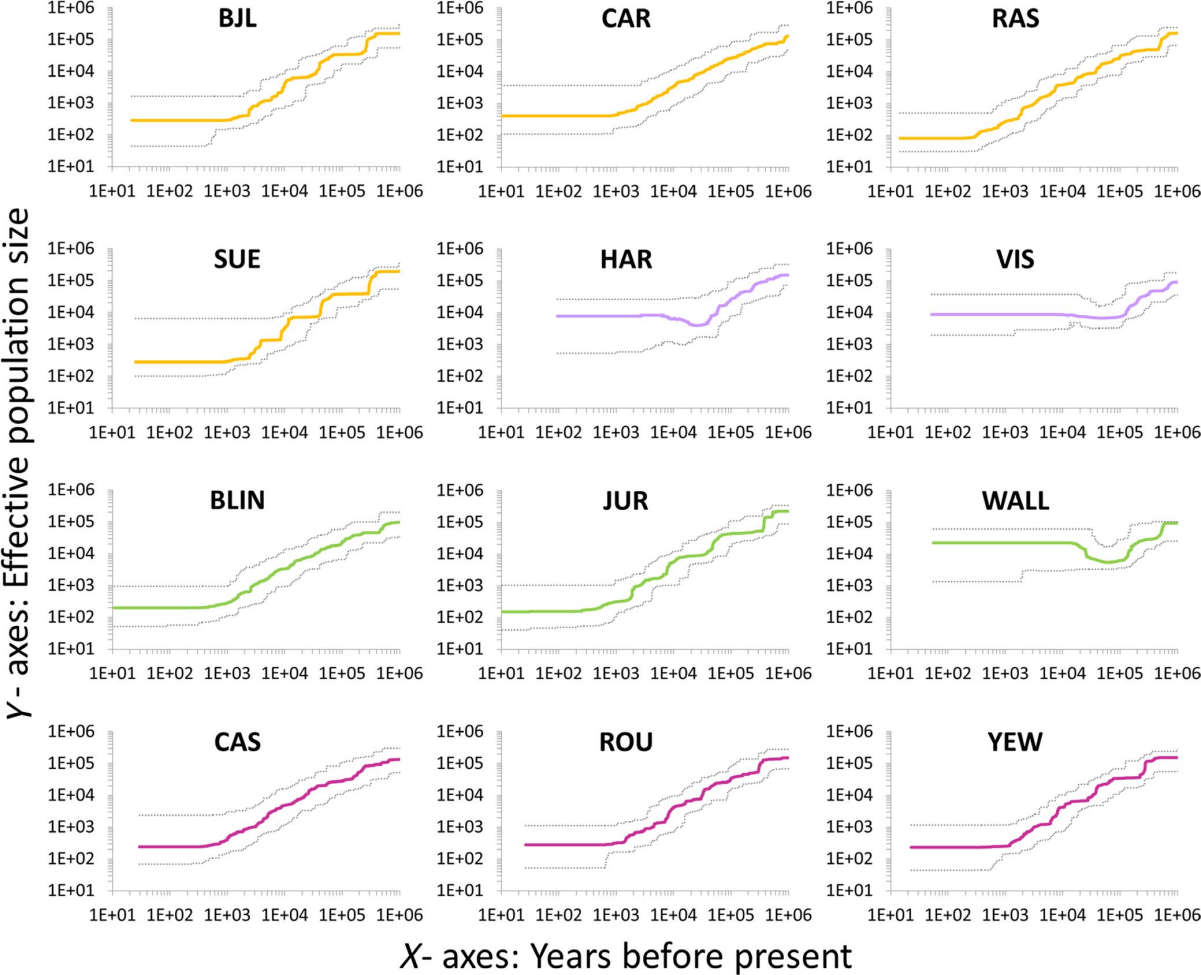
Demographic history of *Taxus baccata* populations inferred with stairway plot (Liu & Fu, 2015). Colored lines show the changes in median effective population size for the last one million years, and dotted lines represent the 95% confidence intervals (2.5% and 97.5% percentiles) based on 200 bootstraps. Populations are colored according to their geographic origin: orange (Iberian Peninsula), green (Switzerland), dark pink (United Kingdom), and lilac (eastern populations: Slovakia and Bosnia‐Herzegovina). Colors are the same as in Figure S6. See Table S2 for details on populations

Demographic inference based on stairway plot revealed a decrease in effective population size for all populations starting around 400,000–600,000 years ago (Figure 4). Since then, the demographic decline has been continuous for populations from the Iberian Peninsula and United Kingdom (Western Europe yew range), as well as for two of the Swiss populations, reaching current effective population sizes (100–400 individuals) around 500–2,000 years ago (Figure 4). The remaining Swiss population (WALL) and those from the Eastern Europe yew range (Bosnia‐Herzegovina, Slovakia), however, seem to have maintained high (7,000–20,000 individuals) and stable effective population sizes during the last 10,000–20,000 years (Figure 4). A higher demographic decline for the former group of populations was also supported by higher positive values of Tajima's *D*, and Fu & Li's D* and F*, indicating stronger demographic bottlenecks (Table 1).

### 3.3 Adaptive variation at SNP, gene, and pathway level

We detected a similar number of significant associations between SNPs and climate with bayescenv and baypass (676 vs. 777), but most of them involved single SNPs per gene. The number of significant associations with at least two different SNPs on the same gene was lower, involving 212/293 SNPs located on 72/111 genes for bayescenv and baypass, respectively. However, only 11 SNPs located on 7 genes had significant associations with the same climatic variables using both methods. At gene level, we found 60 genes with a significantly higher proportion of SNPs associated with climate than expected by chance. Combining these results with those obtained at the SNP level (see Materials and Methods), a total of 11 top candidate genes for climate adaptation were retained (Table 2). These potentially adaptive genes were involved in plant growth, development, endogenous biological rhythms, and several stress responses (Table 2).

**Table 2:** Candidate genes putatively under selection detected for *Taxus baccata*. “Gene level” refers to the number of SNPs detected with Yeaman's et al. (2016) approach (see Materials and Methods for details)

Gene Code (*T. baccata*)	Length (bp)	Nº SNPs	Description	Abbr.	ConGenIE ID (*A. thaliana* best BLAST)	E‐value	Climatic drivers (Period)	Gene level	SNP level	
									BayPass	BayeScEnv
Tbac_18055	2,773	30	Pseudo‐response regulator 7	PRR7	MA_124244g0020 (AT5G02810.1)	0.0	Annual mean precipitation (PRE)	15	2	–
Tbac_19823	1,650	16	P‐loop containing nucleoside triphosphate hydrolases superfamily protein	DNK	MA_892200g0010 (AT1G72040.1)	2.0E‐170	Annual mean precipitation (PRE)	7	2	–
Tbac_129399	843	6	Early‐responsive to dehydration stress protein (ERD4)	ERD4	MA_99251g0010 (AT1G30360.1)	4.3E‐59	Precipitation of the driest month (PRE, LIG)	5 (PRE) 5 (LIG)	4 (PRE) 4 (LIG)	4 (PRE)^a^ 4 (LIG)^a^
Tbac_71106	951	12	TATA‐binding protein‐associated factor BTAF1	BTAF1	MA_79681g0010 (AT3G54280.1)	1.8E‐151	Precipitation of the driest month (PRE, LIG)	7 (PRE) 7 (LIG)	2 (PRE) 3 (LIG)	‐
Tbac_27725	856	19	Plant viral‐response family protein (DUF716)	DUF716	MA_172105g0010 (AT5G13890.3)	9.2E‐111	Annual mean precipitation (PRE)	9	3	–
							Precipitation of the wettest month (PRE)	10	2	–
Tbac_70534	4,869	33	Acetyl‐CoA carboxylase 1	ACC1	MA_38431g0020 (AT1G36160.2)	1.5E‐83	Annual mean temperature (PRE)	11	4	–
Tbac_19996	1,978	42	Putrescine‐binding periplasmic protein‐related	ENF2	MA_10434660g0010 (AT1G31410.1)	0.0	Maximum temperature of the warmest month (PRE)	18	4	8
Tbac_72122	947	16	AAA‐type ATPase family protein	3A‐ATP	MA_8790100g0010 (AT2G45500.2)	5.1E‐59	Maximum temperature of the warmest month (LGM)	7	2	–
Tbac_101255	985	13	Alternative oxidase 1A	AOX1A	MA_10430050g0010 (AT3G22370.1)	5.6E‐141	Annual mean temperature (LGM) Minimum temperature of the coldest month (LGM)	7 7	3 4	1 1
Tbac_101139	1,306	22	Senescence‐associated E3 ubiquitin ligase 1	SAUL1	MA_113300g0020 (AT1G20780.1)	7.6E‐129	Annual mean temperature (LIG)	8	6	–
							Minimum temperature of the coldest month (PRE)	11	8	–
Tbac_70556	6,025	87	Modifier of SNC1	MOS1	MA_958g0010 (AT4G24680.1)	0.0	Minimum temperature of the coldest month (LGM)	38	2	–

Note

LGM, Last Glacial Maximum; LIG, Last Interglacial, PRE, present.

a *Q*‐values < 0.07 for precipitation of the driest month (PRE, LIG); bayescenv also supported a role of annual mean temperature during PRE and LIG (*Q*‐values < 0.05).

**Figure 5:**
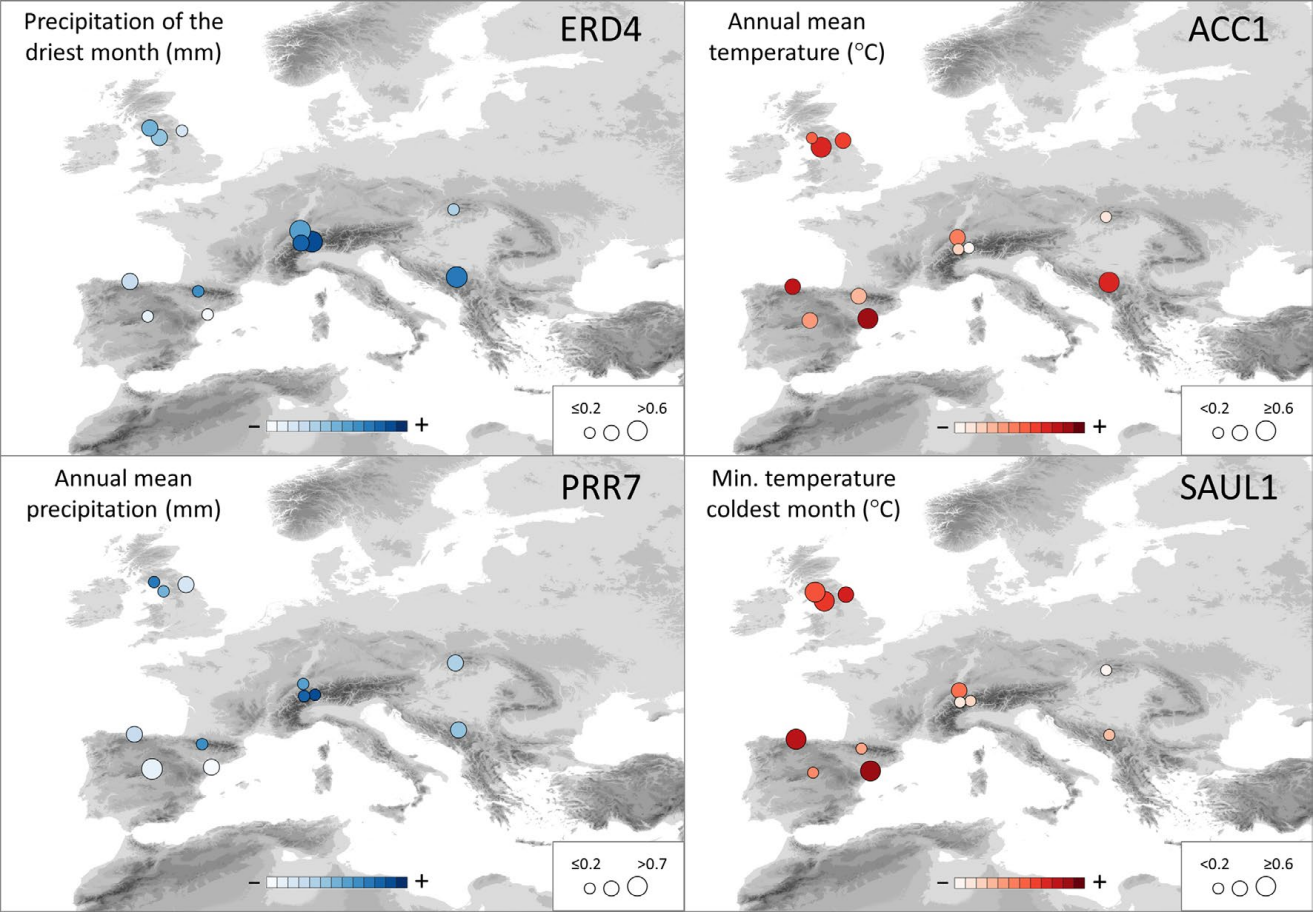
Geographic distribution of minor allele frequency for single SNPs of four top candidate genes and their relation to climate. ERD4: *early‐responsive to dehydration stress protein* (SNP4561, G/A SNP, A allele plotted); PRR7: *pseudo‐response regulator 7* (SNP5227, T/C SNP, C allele plotted); ACC1: *acetyl‐CoA carboxylase 1* (SNP19480, G/C SNP, G allele plotted); SAUL1: *senescence‐associated E3 ubiquitin ligase 1* (SNP346, T/C SNP, C allele plotted). The size of the circle is proportional to allele frequency. Precipitation and temperature variables are shown in blue and red, respectively. Darker colors indicate higher values of climatic variables

The distribution of allelic frequencies in relation to climate of significant SNPs from the 11 top candidate genes confirmed that associations were not due to population structure or demographic processes affecting particular geographic regions (Figure 5 and Figure S6). For instance, for the putative *pseudo‐response regulator 7* gene (PRR7), a significant higher frequency (*F*_1,10_ = 19.13; *p* < 0.01) of the minor allele of SNP5227 was found in those populations with annual mean precipitation below 1,000 mm, irrespectively of their geographic location (Figure 5). The minor allele frequency of SNP4561 from *early‐responsive to dehydration stress* gene (ERD4) increased with precipitation of the driest month, being significantly higher in those populations with minimum precipitation above 60 mm (*F*_1,10_ = 8.50; *p* < 0.05). Minor allele frequencies of SNP19480 (*acetyl‐CoA carboxylase 1*) and SNP346 (*senescence‐associated E3 ubiquitin ligase 1*) increased significantly in those populations with annual mean temperature above 8ºC (*F*_1,10_ = 3.81; *p* = 0.08) and minimum temperature of the coldest month above −3°C (*F*_1,10_ = 62.24; *p* < 0.001), respectively (Figure 5).

Similar numbers of environmental correlations were found for temperature and precipitation variables (six vs. five, respectively). Regarding the climatic period, most of the significant correlations detected involved current climate, although a few were also detected for the last glacial maximum and the last interglacial ones (Table 2).

**Figure 6:**
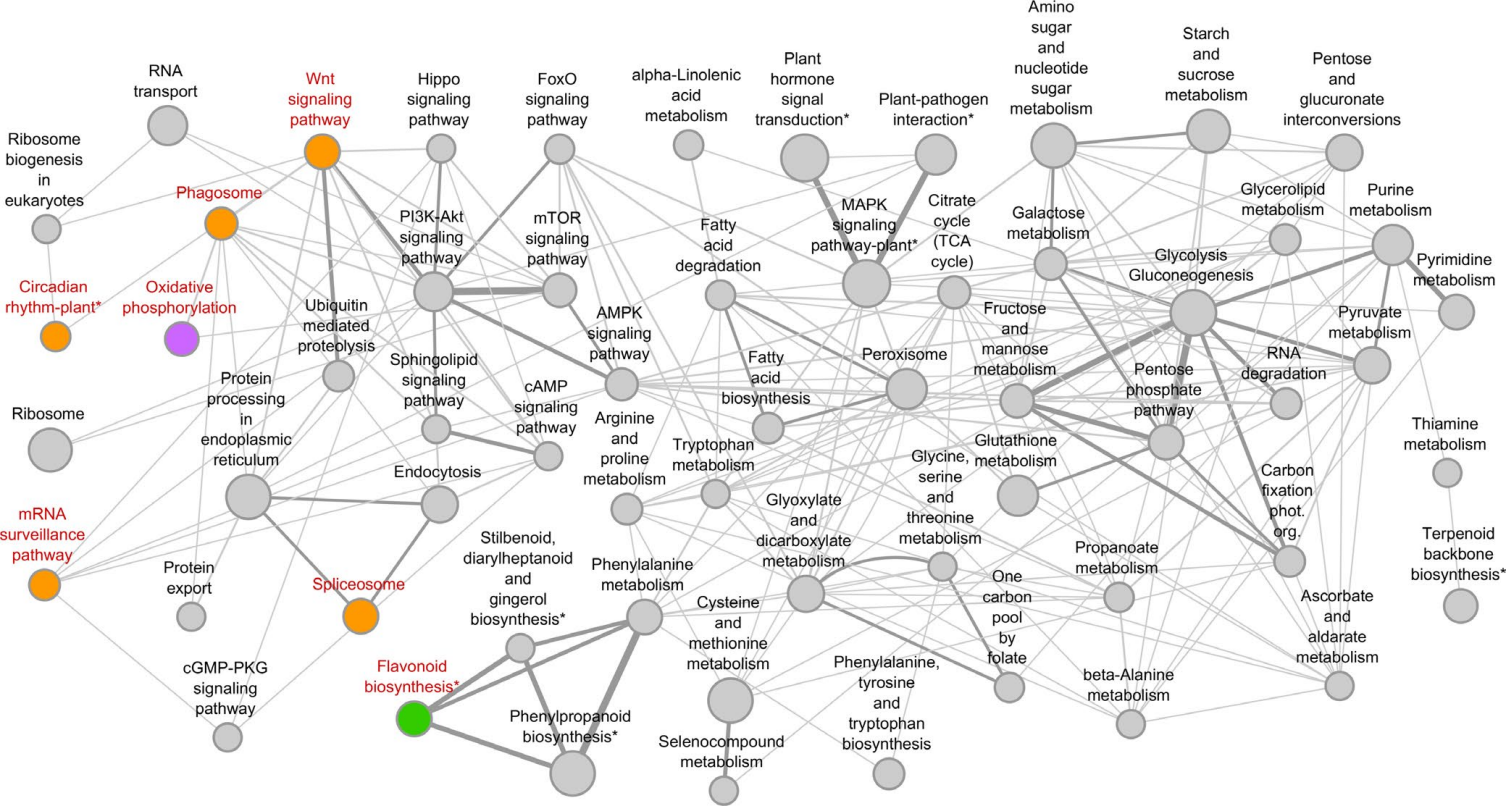
Diagram of the 60 pathways tested for signals of polygenic selection (* indicates pathways that were plant‐specific). The size of the nodes (pathways) is proportional to the number of genes (minimum size = 5, maximum size = 19). Edges represent shared genes between pathways, and edge width is proportional to the number of shared genes (minimum width = 1, maximum width = 7). Candidate pathways for polygenic adaptation are indicated in red. Colored circles indicate significant lower or higher global statistics (*Q*‐values < 0.15; see text and Table 3 for further details). Different colors refer to different statistics: orange to *π*_a_/*π*_s_ (ratio of nonsynonymous to synonymous nucleotide diversity); purple to nucleotide diversity (*π*); green to both *π* and Fu & Li's D*

Only 390 out of 1,128 genes could be mapped on existing KEGG pathway maps for plants. Mapped genes were distributed on 60 distinct pathways, eight of which were plant‐specific (Figure 6). Only 2 of the 390 genes were included among the 11 top candidate genes reported above: The putative *pseudo‐response regulator 7* gene was part of the *circadian rhythm‐plant* pathway, and the putative *acetyl‐CoA carboxylase 1* gene was shared between four different pathways, that is, *fatty acid biosynthesis*, *pyruvate metabolism*, *propanoate metabolism,* and *AMPK signaling* pathway. Interestingly, although the remaining 388 genes that could be mapped on the pathways showed no signs of selection when analyzed individually at the SNP or the gene level, we were able to detect a total of seven pathways that were globally enriched for signals of selection after removing gene redundancy (*Q*‐value < 0.15; Figure 6). Five pathways (*circadian rhythm‐plant*, *mRNA surveillance*, *phagosome*, *spliceosome*, and *Wnt signaling*) showed significantly lower values of *π*_a_/*π*_s_ relative to the total number of pathways analyzed, indicating constrained adaptive evolution (Table 3). This was because many genes (>25%) in these pathways had no variation for nonsynonymous mutations, that is, were highly conserved (Table S5). The *Oxidative phosphorylation* pathway had significantly lower global values of *π*, pointing also to conserved genes, and *flavonoid biosynthesis* exhibited significantly higher values of *π* and Fu & Li's D*, a signature of widespread diversifying selection in this pathway (Table 3; Table S5). None of these seven gene pathways contained genes with extreme values causing highly deviant SUMSTAT scores, but they had significantly lower or higher values because all genes in the pathway globally shifted to small or large values (Table S5).

**Table 3:** Candidate pathways for polygenic adaptation in *Taxus baccata* after removing overlapping genes (“pruning,” see Daub et al., 2013)

Gene pathway	Pathway size before/after pruning	Statistics tested	*p*‐value	*Q‐*value (only values < 0.15)
*circadian rhythm‐plant*	6/5	*π*_a_/*π*_s_	0.005619	0.083
*mRNA surveillance*	7/7	*π*_a_/*π*_s_	0.003249	0.082
*phagosome*	8/6	*π*_a_/*π*_s_	0.004259	0.082
*spliceosome*	10/10	*π*_a_/*π*_s_	0.003789	0.082
*Wnt signaling*	10/10	*π*_a_/*π*_s_	0.000009	0.003
*Oxidative phosphorylation*	9/9	*π*	0.003099	0.073
*flavonoid biosynthesis*	10/10	*π*	0.003259	0.073
*flavonoid biosynthesis*	10/5	FuD*	0.000239	0.011

FuD*: Fu & Li's D* (Fu & Li, 1993).

## 4 DISCUSSION

Our study demonstrates the potential advantage of using multiple complementary approaches for detecting selection to overcome limitations inherent in testing them individually. For instance, the clinal pattern found for phenological traits (i.e., male strobili maturation, proportion of late growth) suggests the existence of local adaptations at the Iberian scale, and this assumption is reinforced by the detection of similar signatures at the European scale, both at the single‐locus (SNP) and multilocus (gene, pathway) levels. Our results also show that polygenic approaches can provide useful and simple tools for detecting signatures of “soft” selection (i.e., acting on multiple genes at the same time). Using this polygenic approach, we were able to identify one biological pathway as potentially under the effect of diversifying selection, when individual approaches such as those focused on single loci failed to detect the signature of selection for any of the genes in this pathway. Finally, our study provides some evidence supporting the contribution of past climate to the potentially adaptive genetic variation observed. Below we discuss these findings within the context of the demography of the species while considering their potential limitations and caveats.

### 4.1 Evidence of selection in response to past and recent climate despite demographic decline

Using extensive genetic resources (1,128 genes and 25,726 SNPs), we confirmed previous demographic studies on English yew that supported the existence of two main Eastern and Western genetic pools across the whole species range (Mayol et al., 2015). In addition, we found a strong signal of demographic decline that was highly consistent across populations, suggesting that common factors may underlie demographic processes in this species and confirming on a wider scale previous reports from the Iberian populations (Burgarella et al., 2012). Although the inferred start of population decline (~400,000–600,000 years ago) was somewhat earlier than that reported by Burgarella et al. (2012), who placed it at ~100,000–300,000 years ago, both studies show the same trend for the species as a whole, which suggests that habitat suitability for English yew has been worsening for the last several millennia, a pattern that is also evident in most European pollen records since the last interglacial period (*c*. 115,000–130,000 year BP; Turner, 2000; de Beaulieu et al., 2001; Koutsodendris et al., 2010). We found, however, that one population from Switzerland and the two from the eastern range (Bosnia‐Herzegovina, Slovakia) were characterized by stable and larger population sizes, which is in agreement with the pattern of decreasing genetic diversity from east to west reported for English yew, and could be explained by the milder climatic conditions experienced by easternmost populations since the long past (Mayol et al., 2015).

Interestingly, despite the small current effective population size inferred for most populations, we found evidence for potentially adaptive variation at both the phenotypic and molecular level. Following theoretical considerations, Kawecki and Ebert (2004) suggested that convincing evidence for local adaptation requires testing for population × habitat interaction in reciprocal transplant experiments. However, even a single common garden experiment can help to reveal genetic differentiation among widely distributed, divergent populations, providing indirect evidence of past adaptation (Cheplick, 2015). In our study, the differential growth (a proxy for fitness in forest trees) of the Iberian yew populations was associated with the climatic distance between the site of origin and the conditions experienced in the common environment, suggesting local adaptation. Similarly, the among‐population variability in shoot elongation and male strobili maturation, both linked to temperature clines, also pointed to the selective effects of climate on phenological patterns. Compared to more temperate coastal populations, those from continental environments (colder and subject to more pronounced temperature variations) showed both faster growth and earlier maturation of male strobili at the trial site, which again suggests that natural selection has favored genotypes optimized to the short time frame available for successful growth and reproduction. Of course, part of the observed variability among genets and populations could also be due to strong maternal clone effects (C‐effects, see Burdon & Shelbourne, 1974). These transgenerational clonal effects have been widely reported (see review in Schwaegerle, Mcintyre, & Swingley, 2000). However, experimental treatments in plants suggest that the magnitude of C‐effects usually vanishes with time, particularly for late developing traits (e.g., mass or growth; Libby & Jund, 1962; Schwaegerle et al., 2000). Therefore, while C‐effects cannot be fully ruled out, their importance is expected to be relatively low, since measures of plant performance were obtained from plants growing on a common environment for 10–15 years. Many studies on tree species have also reported clinal patterns of phenotypic differentiation, in particular for phenological and fitness‐related traits, suggesting that tree populations are locally adapted over large parts of their native ranges (see review in Savolainen, Pyhäjärvi, & Knürr, 2007; Alberto et al., 2013). Our findings for English yew are similar to these studies but, unlike many widespread “model” tree species, adaptation occurs in the face of limited effective population sizes and restricted gene flow (see Dubreuil et al., 2010).

Analyses at the molecular level also suggested local adaptation for phenological traits, since significant signatures of selection were found at the European scale for both the *circadian rhythm* pathway (discussed further in the next section) and the putative *pseudo‐response regulator 7* gene (PRR7). In *A. thaliana*, PRR7 is an essential component of the circadian clock that is directly associated with developmental transitions such as flowering (Nakamichi et al., 2007), and putative orthologous genes have been identified in *Picea abies* (L.) Karst. (Gyllenstrand et al., 2014). In this conifer, PRR7 shows partly conserved protein domains and displays diurnal rhythms of expression similar to those observed in *Arabidopsis*, although important differences exist between angiosperms and gymnosperms clock systems (Gyllenstrand et al., 2014).

Interestingly, a significant association with latitude has been reported for candidate SNPs at the PRR7 gene in European populations of Norway spruce (Chen, Källman, et al., 2012), in accordance with the pattern detected for English yew and annual mean precipitation at the European scale. Although most studies investigating the adaptive response of circadian genes have focused on the role of temperature and photoperiod, recent studies in *Arabidopsis* indicate that daily humidity oscillations are also involved in the regulation of specific circadian outputs besides those co‐regulated with the light–dark cycle (Mwimba et al., 2018). Moreover, the PRR7 gene seems to be directly involved in the regulation of both cold and drought stress responses of plants in coordination with an ABA‐dependent mechanism (Liu, Carlsson, Takeuchi, Newton, & Farré, 2013). This could explain the apparent contradiction between the results obtained in the common garden, that probably reflect the overall response of the circadian clock genes to temperature, and the variation found for the PRR7 gene at the European scale in response to precipitation.

Apart from PRR7, several other candidate genes relevant for forest tree adaptation were detected in our study (Table 2). Some of these genes are rapidly activated in response to drought stress (*early‐responsive to dehydration stress* (ERD4): Alves & Fietto, 2013), cold acclimation (*acetyl‐CoA carboxylase 1* (ACC1): Amid, Lytovchenko, Fernie, Warren, & Thorlby, 2012; *Alternative oxidase 1A* (AOX1A): Fiorani, Umbach, & Siedow, 2005; *Putrescine‐binding periplasmic protein‐related* (ENF2): Cuevas et al., 2008), and pathogen effectors (*Modifier of SNC1* (MOS1): Li, Tessaro, Li, & Zhang, 2010), while others are central components of the regulatory network controlling leaf senescence in plants (*senescence‐associated E3 ubiquitin ligase 1* (SAUL1): Lee et al., 2016). However, despite the candidate genes reported in this study provide a first set of genes to further explore English yew's adaptive responses at the molecular level, there is a need to confirm our results using a higher number of samples, as well as to provide unequivocal links between allelic variation and fitness before concluding that the patterns found are truly adaptive. Both theoretical and empirical studies suggest that accurate estimates of genetic differentiation can be obtained with a large number of SNPs (≥1,500) even at small sample sizes (Nazareno, Bemmels, Dick, & Lohmann, 2017; Willing, Dreyer, & Van Oosterhout, 2012) but, in some cases, detection of regions under selection may be affected by nonrandom processes such as population structuring. Detecting the same patterns in a larger number of samples could help to circumvent these problems and to increase the confidence in our results.

Although most of the variation found for these candidate genes was correlated to present climate, we also were able to detect significant associations with past climate for a few genes (Table 2). It must be noted, however, that our approximation to detect the potential contribution of past climate is not free of uncertainty, since it is based on the assumption that current distribution of English yew has been similar across the different Quaternary periods. On the other hand, the severe bottleneck that the populations have undergone since the long past probably has contributed to the loss of variants, making more difficult the detection of signatures of selection in response to past climate. Despite these potential caveats, there is some evidence suggesting that some of the associations found for allelic variation and past climates could be adaptive. For instance, none of the associations found for the current climate were also found for the LGM. The few genes (three) whose variation was found to be linked to more than one climatic period involved both interglacials (PRE, LIG), with two of them also associated with the same environmental variable (i.e., precipitation of the driest month; Table 2). This could just reflect a higher climatic similarity between the present day climate and that of the last interglacial, but also points to a differential response to glacial and interglacial climates. Additional evidence is provided by the lack of correlation between allelic variation and precipitation variables during LGM (see Table 2), suggesting that water availability could have acted as a more important selective pressure during interglacials than during glacial periods. In contrast, similar temperature‐mediated responses were found for cold and warm glacial episodes, since the same number of candidate genes involved in thermal adaptation was detected for the glacial (LGM) and interglacial (PRE, LIG) periods. However, further exploration of the relationship between adaptive variation and past and current climates in other tree species will be needed to fully test this hypothesis.

### 4.2 Signature of polygenic adaptation in biological pathways

In addition to single‐locus methods, we also applied a gene set enrichment test that has been used successfully to detect polygenic adaptation in the human genome (Daub et al., 2013, 2017; Foll et al., 2014). With this approach, we found seven biological pathways that were globally enriched for signals of selection, being therefore good candidates for polygenic adaptation. Five pathways involved in essential primary functions that are critical for plant survival, namely catabolism (*phagosome*), genetic information processing (*mRNA surveillance*, *spliceosome*) or environmental information processing (*circadian rhythm*, *Wnt signaling*), were characterized by highly conserved genes (that is, significantly lower overall values of the ratio of nonsynonymous to synonymous nucleotide diversity). In fact, many of the constituent genes of these pathways showed no variation for nonsynonymous mutations (Table S5), suggesting that they evolved under rigorous functional or structural constraints. An additional pathway, which is involved in crucial energy metabolism processes (*Oxidative phosphorylation*), showed significant lower overall values of nucleotide diversity. These results are consistent with the expected prevalence of purifying selection for genes and/or genomic regions of functional relevance, especially for nonsynonymous sites (Wright & Andolfatto, 2008), driving a global shift toward low values of nucleotide diversity and/or high efficacy of selection (that is, low *π*_a_/*π*_s_).

Within this group, however, the *circadian rhythm* pathway deserves special attention (Figure S7). Despite the prevalent role of purifying selection suggested by the gene enrichment approach for the whole pathway, the PRR7 gene seems to be a good candidate for adaptive variation in response to climate in English yew, as discussed in the previous section. These results are highly consistent with those found in *Picea abies*, where both purifying and diversifying selection have been invoked to explain the diversity found for genes in the photoperiodic (*circadian rhythm*) pathway (Chen et al., 2016). In this species, *upstream* light receptor genes, such as *cryptochrome‐1*, *phytocromes,* and *Zeitlupe*, have about half the nucleotide diversity than the *downstream* genes of the circadian clock, like *Gigantea* and the *pseudo‐response regulators 1, 3,* and *7* (*π* = 0.0016 vs. *π* = 0.0031, respectively; Källman et al., 2014). Accordingly, the signals of diversifying selection are mostly identified in *downstream* genes (Chen, Källman, et al., 2012; Chen et al., 2016; Källman et al., 2014). These results are highly consistent with our findings for *T. baccata* (Table S5), where the nucleotide diversity for the putative PRR7 gene (*π* = 0.0036) was 3‐fold that of the remaining genes (*π* = 0.0011). In addition, the ratio of nonsynonymous to synonymous variation for the PRR7 gene was between 5–30 times greater than that of the other genes in this pathway, suggesting that functional constraints for this particular gene might be much more relaxed (Table S5). In fact, results for both *P. abies* and *T. baccata* are in agreement with the prediction that selective constraint is progressively relaxed along metabolic pathways because *upstream* genes might have greater pleiotropic effects than *downstream* ones, since *upstream* genes are required for a wider range of end products (Cork & Purugganan, 2004), as has been proven in other metabolic pathways in plants (e.g., *carotenoid biosynthesis* in carrot, Clotaut, Peltier, Soufflet‐Freslon, Briard, & Geoffriau, 2012; *isoflavonoid* pathway in soybean, Chu, Wang, Cheng, Yang, & Yu, 2014; but see Olson‐Manning, Lee, Rausher, & Mitchell‐Olds, 2013 for *glucosinolate* pathway in *Arabidopsis thaliana*).

Finally, the *flavonoid biosynthesis* pathway had significantly higher values for overall nucleotide diversity (*π*) and Fu & Li's D*, pointing to divergent selection acting across English yew's geographic range. Flavonoids are plant secondary metabolites that are involved in a wide array of biological functions, including protection against ultraviolet (UV) radiation and phytopathogens, signaling during nodulation, pollen fertility, auxin transport and coloration of flowers, fruits and seeds (Falcone Ferreyra, Rius, & Casati, 2012). Interestingly, neither analysis at the SNP nor at the single‐gene level showed significant signatures of selection for any of the genes in this pathway. Thus, our approach of testing for selection at the pathway level may have allowed us to identify genetic responses to the local environment as a whole, not only to climate (as in the SNP/gene‐level approaches) and/or better assess signatures of selection for highly polygenic adaptive traits. Several genes with small effect mutations can together have a large impact on a biological pathway and, as stress‐responsive genes, those coding for flavonoids are expected to evolve under the distinct selection pressures of each local environment, including day length, light quality, water availability and temperature, as well as local pathogens. It is thus possible that the higher overall nucleotide diversity and Fu & Li's D* values reflect slight changes of allele frequency but at many genes at the same time, which is difficult to identify with conventional single‐locus approaches (Pritchard et al., 2010). This opens a promising line of research to be applied to other nonmodel forest trees.

### 4.3 Concluding remarks

Our rangewide study in English yew using a large number of SNP markers allowed us to confirm an overall demographic history characterized by two gene pools in Western and Eastern Europe, with an admixture zone in Central Europe, and population decline in most populations, extending initial evidence based on nuSSRs in Burgarella et al. (2012). Interestingly, despite demographic decline, we found evidence of local adaptation to climate both for phenotypic traits in a common garden and SNPs from several candidate genes, probably because levels of nucleotide diversity (*π* = 0.00437) are still large in this long‐lived tree. Several new candidate genes for positive and negative selection in forest trees were identified or confirmed in this study, and an approach to detect potential polygenic selection in biological pathways was successfully tested. In particular, we identified the *flavonoid biosynthesis* pathway as a general stress‐response pathway that deserves further attention, including genomic studies in other plant systems. Our study also suggests that the integration of complementary information coming from different sources could be useful to detect selection in nonmodel species for which common gardens and genomic resources are limited. Such an approach can also help the field to advance beyond correlational “black‐box” methods (i.e., “reverse ecology”) to systems biology‐driven ones, which are more informative in terms of drivers and mechanisms of adaptation. Finally, our study also contributed to the emerging view that while adaptation to current climates explains part of the contemporary distribution of standing genetic variation, it is also essential to consider adaptation to past climates, especially for long‐lived forest trees. Understanding the drivers and mechanisms of adaptation is particularly relevant for highly threatened species such as the English yew, which is protected by environmental laws in several European countries, and may help to develop sound conservation programs and reforestation schemes.

## CONFLICT OF INTEREST

None declared.

## Data Availability

Data for this study are available at Dryad Digital Repository: ://doi.org/10.5061/dryad.78j501j (Mayol et al., 2019).
