# Supplementary material for: A multiscale approach to detect selection in nonmodel tree species: Widespread adaptation despite population decline in Taxus baccata L
Source: Evol Appl. 2019 Jul 19;13(1):143–60. doi: 10.1111/eva.12838 (PMC6935595; doi:10.1111/eva.12838)
Supplement: Supplementary file 1 [file EVA-13-143-s001.docx]

**SUPPLEMENTARY MATERIALS**

Additional Supporting Information may be found online in the supporting information tab for this article.

Supplementary Tables S1 to S4 are provided in a separate excel file.

**FIGURE S1**. Estimated female and male marginal means according to the mixed model for the analysis of shoot growth (volume, mm^3^; see methods for details). Bars are standard errors.

**FIGURE S2**. Variation among populations and years in shoot growth (volume, mm^3^). Values are estimated marginal means (see methods section for details). Bars are standard errors.

**FIGURE S3**. Variation among populations and years in proportion of late (summer and fall) growth in length. Values are estimated marginal means (see methods section for details). Bars are standard errors.

**FIGURE S4**. Estimated female and male marginal means in phenological patterns of shoot development across years. Values are estimated marginal means the proportion of late (summer and fall) shoot growth in length (see methods section for further details). Bars are standard errors.

**FIGURE S5**. Variation among populations and years in male cone maturation (ratio open to total cones). Values are estimated marginal means (see methods section for details). Bars are standard errors.

**
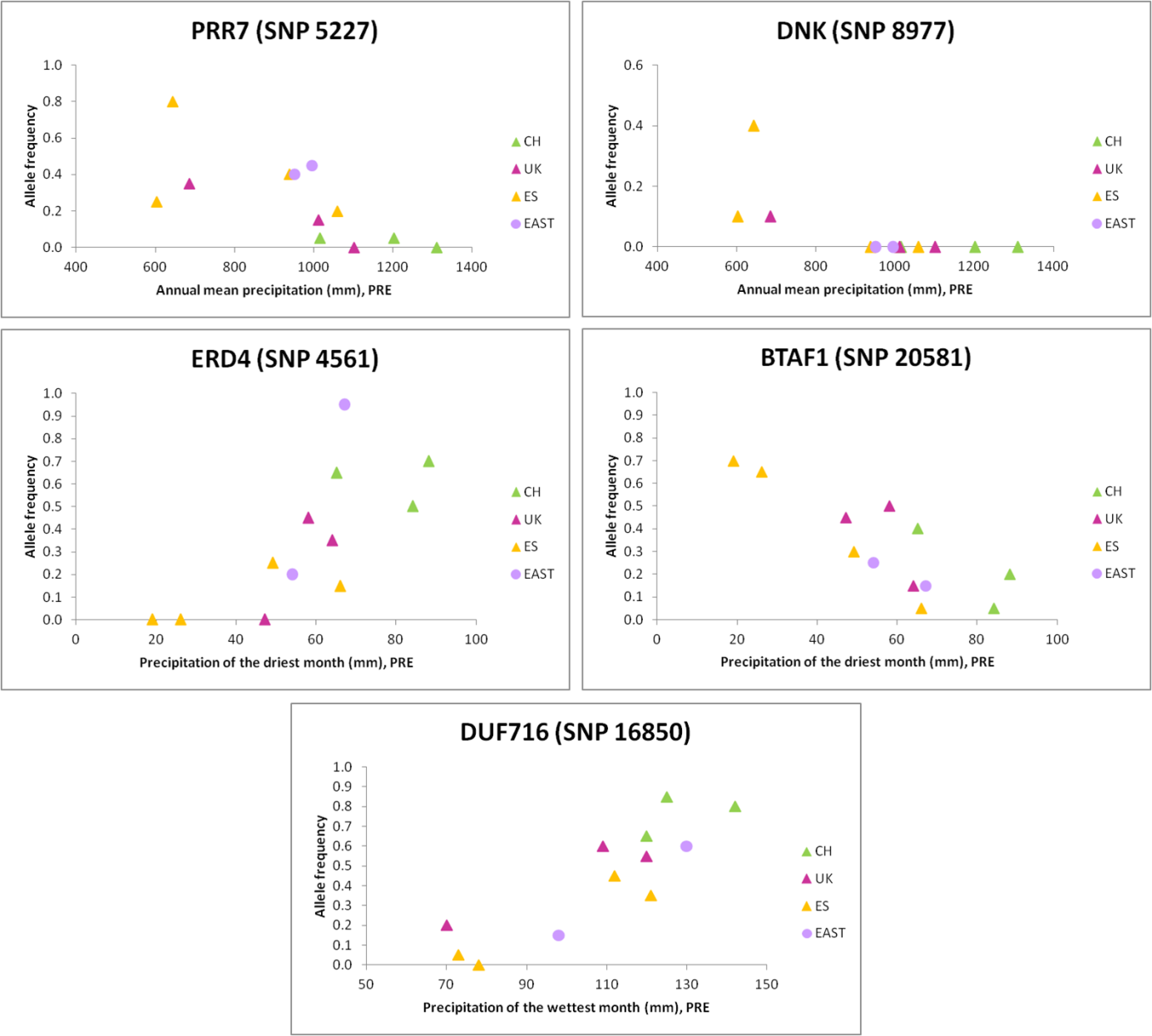
**

**FIGURE S6**. Minor allele frequency distribution of some SNPs from top candidate genes in relation to precipitation. CH: Switzerland; UK: United Kingdom; ES: Spain; EAST: Slovakia and Bosnia-Herzegovina*. PRR7=Pseudo-response regulator 7; DNK=Deoxyribonucleoside kinase*; ERD4=*Early-responsive to dehydration stress protein ERD4*; BTAF1=*TATA-binding protein-associated factor BTAF1*; DUF716=*Plant viral-response family protein DUF716*.


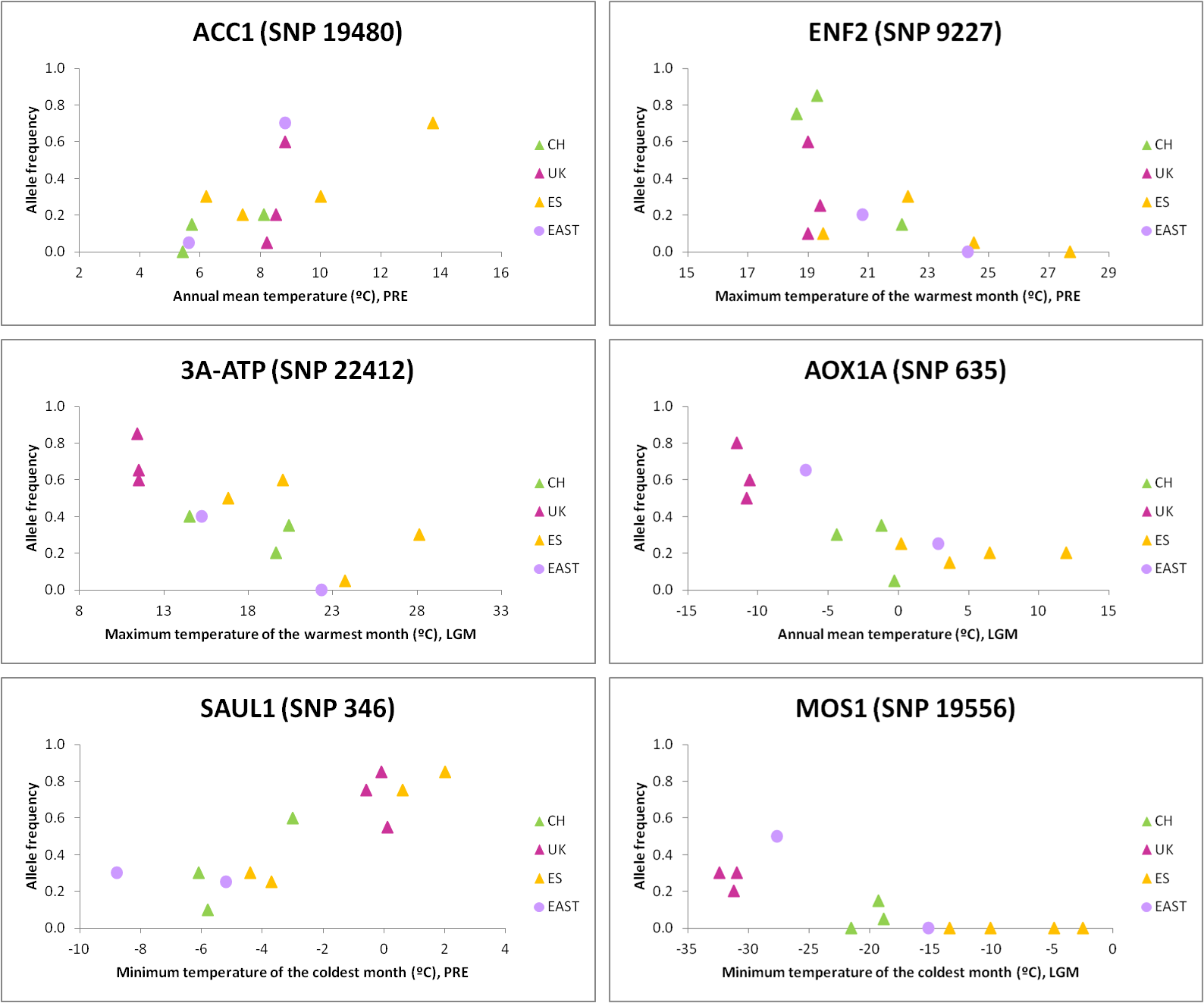


**FIGURE S6 (cont.)**. Minor allele frequency distribution of some SNPs from top candidate genes in relation to temperature. CH: Switzerland; UK: United Kingdom; ES: Spain; EAST: Slovakia and Bosnia-Herzegovina. ACC1=*Acetyl-CoA carboxylase 1*; ENF2=*Putrescine-binding periplasmic protein-like protein*; 3A-ATP=*AAA-type ATPase family protein*; AOX1A=*Alternative oxidase 1A*; SAUL1=*Senescence-associated E3 ubiquitin ligase 1*; MOS1=*Modifier of SNC1*.


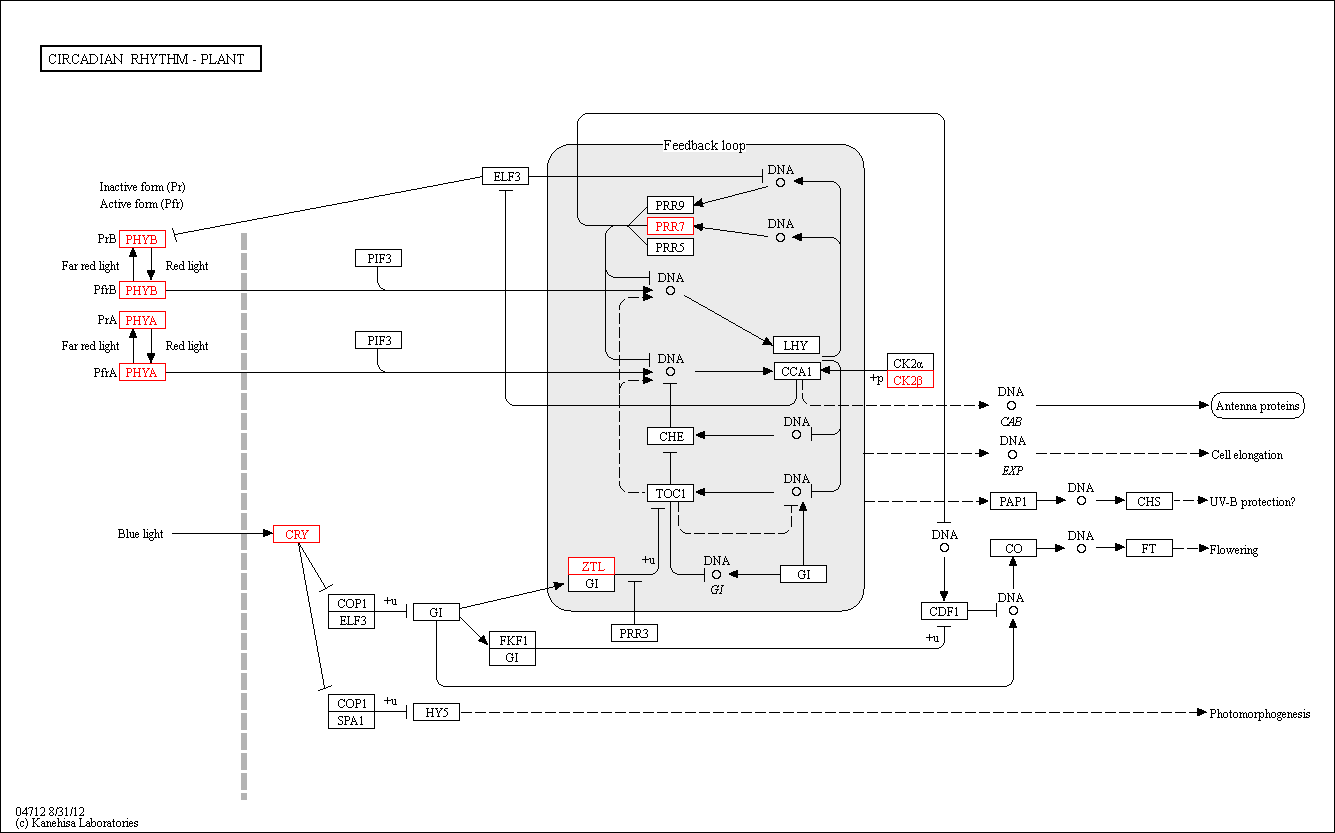


**FIGURE S7**. Circadian rhythm-plants pathway (downloaded from KEGG). The six genes sequenced in this study are shown in red. CRY1: cryptochrome-1; CSNK2B: casein kinase II subunit beta; PHYA: phytochrome A; PHYB: phytochrome B; PPR7: pseudo-response regulator 7; ZTL: clock-associated PAS protein Zeitlupe.
